# A multicenter retrospective controlled study of the Pipeline™ and Tubridge™ Flow Diverter devices for intracranial wide-necked aneurysms

**DOI:** 10.3389/fneur.2022.1014596

**Published:** 2022-10-13

**Authors:** Heng Cai, Fangyu Yang, Yousong Xu, Yu Geng, Jinwei Li, Yugang Li, Kailei Fu, Chang Liu, Meiyan Wang, Zhiqing Li

**Affiliations:** ^1^Department of Neurosurgery, Shengjing Hospital of China Medical University, Shenyang, China; ^2^Department of Neurosurgery, General Hospital of Northern Theater Command, Shenyang, China; ^3^Department of Neurosurgery, First Affiliated Hospital of Dalian Medical University, Dalian, China; ^4^Department of Interventional Radiology, Liaohe Oilfield General Hospital, Panjin, China; ^5^Department of Stroke Center, First Hospital of China Medical University, Shenyang, China; ^6^Department of Neurology, The First Hospital of China Medical University, Shenyang, China; ^7^Department of Neurosurgery, The First Hospital of China Medical University, Shenyang, China

**Keywords:** intracranial wide-necked aneurysm, flow diverter devices, coil, endovascular therapy, perioperative complications

## Abstract

**Purpose:**

To compare the safety and efficacy of Pipeline^TM^ and Tubridge^TM^ Flow Diverter devices (FDs) in the treatment of intracranial wide-necked aneurysms.

**Methods:**

We retrospectively analyzed the clinical data of 92 patients with intracranial wide-necked aneurysms who were treated with those two flow-diverter devices (FDs) at four participating centers between July 2012 and December 2020.

**Results:**

This study included 92 patients who underwent endovascular therapy using either Pipeline™ (*n* = 39) or Tubridge^TM^ (*n* = 53) for treating intracranial wide-necked aneurysms. The periprocedural complication developed in 2.56% (1/39) patients of Pipeline group and 3.77% (2/53) patients of the Tubridge^TM^ group. During perioperative period, one patient in Pipeline™ group showed subarachnoid hemorrhage (2.56%, 1/39) and two ischemic complications in the Tubridge™ group (3.77%, 2/53). Follow-up assessments were conducted on 31 patients (79.49%) in the Pipeline™ group (the mean follow-up period was 9.7 ± 3.3 months). The rate of complete aneurysm occlusion at the final angiographic follow-up was 77.42%. Patients with a modified Rankin scale (mRS) score of 0.44 ± 0.31. Follow-up assessments were conducted on 42 patients (79.25%) in the Tubridge^TM^ group (the mean follow-up period was 9.1 ± 4.4 months). The rate of complete aneurysm occlusion at the final angiographic follow-up was 85.71%. Patients with mRS score of 0.52 ± 0.28. Three patients showed parent artery stenosis, and one showed parent artery occlusion.

**Conclusion:**

Both the Pipeline^TM^ and Tubridge^TM^ are safe and effective for the treatment of intracranial wide-necked aneurysms, with no significant difference in the rate of complete aneurysm occlusion and perioperative complications between the two FDs.

## Introduction

The results of the International Subarachnoid Aneurysm Trial published in 2005 identified endovascular therapy as one of the primary treatments for intracranial aneurysms (1, 2). However, the rate of complete occlusion in endovascular treatment of wide-necked aneurysms remains suboptimal, and the risk of long-term recurrence remains high (3, 4).

Over the past decade, flow-diverter devices (FDs) have emerged as first-line endovascular treatment devices for intracranial aneurysms, particularly large and giant aneurysms (5, 6). The pipeline embolization device (PED) (Pipeline^TM^; Medtronic Inc, Dublin, Ireland) was the first FD approved for clinical use and is currently the most reliable FD with respect to clinical and laboratory evidence (7–10). The Tubridge^TM^ vascular reconstruction device (TB) (Tubridge™; MicroPort Medical Company, Shanghai, China) was the first FD developed in China (11). We conducted a multicenter retrospective study comparing the clinical efficacy of the PED and TB for the treatment of intracranial wide-necked aneurysms and evaluated the prognosis of patients during the follow-up period. This study provides a theoretical basis for individualized selection of FDs on the basis of the characteristics of the patient's aneurysm.

## Materials and methods

### Participants

The clinical data of patients with unruptured intracranial wide-necked aneurysms confirmed by computed tomography angiography (CTA)/magnetic resonance angiography (MRA)/digital subtraction angiography (DSA) and treated with FDs at four medical centers (27 cases at the former General Hospital of Northern Theater Command, Shenyang, China; 27 at Shengjing Hospital of China Medical University, Shenyang, China; 10 at Liaohe Oilfield General Hospital, Panjin, China; and 28 at First Affiliated Hospital of Dalian Medical University, Dalian, China) between January 2012 and December 2020 were retrospectively analyzed. Patients were divided into two groups based on the FD implanted: The PED group included patients treated with the PED (*n* = 39, preoperative baseline modified Rankin scale [mRS] score of 1.15 ± 0.34), while the TB treatment group included patients treated with the TB (*n* = 53, preoperative baseline mRS score of 1.20 ± 0.29). An intracranial wide-necked aneurysm was defined as an aneurysm with a neck width of ≥4 mm (absolute wide-necked aneurysm) and/or dome-to-neck ratio ≤2 (relative wide-necked aneurysm).

The study cohort included 38 male and 54 female patients (range, 43–76 years; mean age, 59.7 ± 8.9 years). Forty-nine cases (53%) showed concomitant primary hypertension, and 44 (48%) showed concomitant type II diabetes mellitus. Clinical symptoms included headache (*n* = 38), focal neurological deficits included oculomotor paralysis, blurred vision et al. (*n* = 29), TIA:transient ischemic attack (TIA) (*n* = 6), and epilepsy (*n* = 1); 18 cases showed no clinical symptoms. The location of aneurysm included the ophthalmic segment of the internal carotid artery (ICA) in 20 cases, the cavernous segment of the ICA in 28, the paraclinoid segment of the ICA in 20, the posterior communicating segment of the ICA in 17, the petrous segment of the ICA in five, and the vertebrobasilar artery in two. All aneurysms in the group were saccular aneurysms, with a mean diameter of 6–30 mm (14.6 ± 4.5 mm); 14 cases involved aneurysms with a diameter of ≥ 25 mm, and 47 involved aneurysms with a diameter of <10 mm. The baseline characteristics of analyzed patients were showed in Table 1.

**Table 1:** Baseline characteristics of analyzed patients.

	**Pipeline**	**Tubridge**	***P***
**Number of cases**	39	53	
**Age(mean ±SD years)**	58.8 ± 7.7	60.1 ± 9.1	0.2831
**Baseline mRS score (mean ±SD)**	1.15 ± 0.34	1.20 ± 0.29	0.2845
**Gender**			0.8304
male	17	21	
female	22	32	
Hypertension	19	30	0.528
Diabetes	18	26	0.8347
**Clinical presentation**			0.439
Headache	18	20	
Neurological deficit	14	15	
TIA	1	5	
Epilepsy	0	1	
Asymptomatic	6	12	
**Aneurysm location**			
ICA ophthalmic	8	12	
ICA cavernous	13	15	
ICA paraclinoid	8	12	
ICA communicating	7	10	
ICA petrous	2	3	
vertebrobasilar artery	1	1	
**Aneurysm size (mm)**			0.7753
<10 mm	19	28	
10 mm−25 mm	14	17	
≧25 mm	6	8	

TIA, transient ischemic attack; ICA, internal carotid artery.

### Perioperative treatment strategy

The preoperative antiplatelet therapy strategy was selected as described in the literature. All patients were given either oral clopidogrel 75 mg + aspirin 100 mg (≥3 d) or clopidogrel 75 mg + aspirin 300 mg (≥1 d) continuously before surgery. Postoperatively, dexamethasone 10 mg was administered intravenously for 3 d, followed by oral clopidogrel 75 mg + aspirin 100 mg q.d. (≥3 months), which was then changed to a single oral antiplatelet drug. Routine head CT re-examination was performed on postoperative day 2. Antiplatelet drugs were immediately discontinued if the patient developed subarachnoid hemorrhage in the perioperative period.

### Treatment method

All procedures were performed under general anesthesia. The femoral artery was punctured using a modified Seldinger technique. Bilateral femoral artery access was used in 87 patients, and unilateral femoral artery access was used in five.

First, complete cerebral angiography and three-dimensional (3D) vascular reconstruction were performed to elucidate the relationship of the aneurysm location, size, and neck width with the artery containing the aneurysm. Neck compression angiography was used to elucidate the status of vascular compensation in anterior circulation aneurysms. The working angle was selected by 3D imaging; a 6F long sheath or 8F guiding catheter was used; and a 6F Navien guiding catheter (Medtronic, USA) was placed into the target artery under the guidance of a guidewire. A Synchro14 200 microwire was used to guide the SL-10 microcatheter across the neck of the aneurysm to the most distal normal vessel possible under a path diagram. The Synchro microwire was withdrawn and replaced with a Transend 300 microwire to the site, and the Marksman microcatheter/T-track microcatheter was replaced to the most distal part of the aneurysm. A 6F guiding catheter was placed in the sheath of the contralateral femoral artery to reach the aneurysm donor artery, and a microwire was used to guide the SL-10 microcatheter into the aneurysm. The appropriate FD was selected and delivered to the target location; the head end of the microcatheter was placed in the distal flat vessel; and the stent was released after confirming the fit such that it completely covered the aneurysm neck. Next, the stent was released, and angiography was performed again to determine whether the artery with the aneurysm and branching and perforating vessels were patent. The procedure was ended with natural neutralization of heparin by preplaced microcatheterization with coil filling and intra-aneurysmal contrast retardation. The indication for using of the coil in this study were considered that preventing the FD herniate into the aneurysm or three-dimensional rotational angiography showed that blood jet persistent inflow jet impingement to the aneurysm. FD implantation was immediate; microwire massage was not performed in 12 early cases, while subsequent microwire massage techniques were performed in the next 80 cases as follows: repeated passage through the FD using the microwire collaterals formation technique, combined with repeated passage of the microcatheter for microwire massage when necessary, which contributed to better opening and apposition of the FD. In some cases, balloons were applied to dilate the proximal end of the FD, and the incidence of ischemic complications was substantially reduced. During application, we also found less change in the canal diameter after PED release and a lower rate of shortening; the TB showed adaptability to different vessel diameters and a significantly higher rate of shortening. In all cases, angiography was performed after microwire massage, and proximal balloon dilation was required in 11 cases. This was performed as follows: if the opening was still unsatisfactory after FD release or if the proximal end of the stent was poorly apposed to the wall causing blood flow to still shoot proximal to the aneurysm, proximal balloon dilation was performed.

### Imaging and clinical evaluation

Regular postoperative follow-up was conducted, and whole-brain DSA re-examination was performed 6–12 months after surgery and graded as complete occlusion, cervical residual near-complete occlusion, or incomplete occlusion. The prognosis of patients was evaluated using the mRS. Technical complications were defined as complications that occurred intraoperatively due to surgical operations. Perioperative clinical complications were defined as symptomatic complications that occurred during the perioperative period, excluding technical causes and including worsening of original symptoms, subarachnoid hemorrhage, cerebral infarction, and focal neurological deficits. All follow-up evaluations were performed using cerebral angiography re-examination to assess aneurysm healing and blood flow in the aneurysm-carrying artery, as well as using mRS scores (12) to evaluate patients' recovery of neurological function at the follow-up endpoint and to record deaths.

### Statistics

SPSS 25.0 software was used for statistical analyses of data. Measurement data conforming to a normal distribution were expressed as means ± standard deviations, and the *t*-test was used for comparisons between groups. Measurement data not conforming to a normal distribution were expressed as medians and quartiles (M [P_25_, P_75_]), and the rank-sum test was used for comparisons between groups. Count data were expressed as the number of cases and percentage (cases [%]), and the χ^2^ test or Fisher's exact probability method was used for comparisons between groups. Differences with *p* < 0.05 were considered statistically significant.

## Results

### Intraoperative status

**Figure 1:**
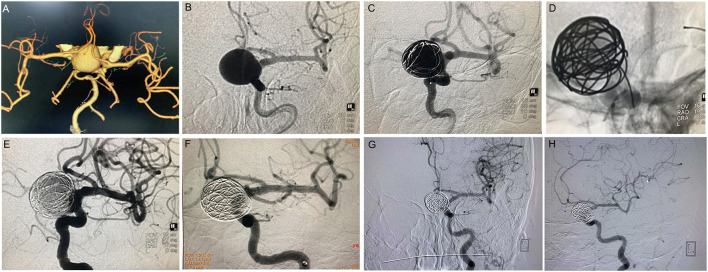
Pipeline^TM^ treatment of giant wide-necked aneurysm in the clinoid segment of the internal carotid artery. **(A)** CTA results show a giant wide-necked aneurysm in the clinoid segment of the left internal carotid artery; **(B)** working angle measurement indicates an aneurysm size of approximately 19.2 mm × 22.7 mm and an aneurysm neck of 15 mm; **(C)** first, a microcatheter is used to release the coil, and the coil is formed into a basket to support Pipeline^TM^ release; **(D)** release of the 4.0 × 25 Pipeline^TM^; **(E)** the micro-guide wire is used to massage the stent to make the stent fit the wall completely; **(F)** 30 min later, the aneurysm is observed to be completely unremarkable on angiographic re-examination; and **(G,H)** 6 months after the procedure, DSA imaging re-examination shows complete healing of the aneurysm.

**Figure 2:**
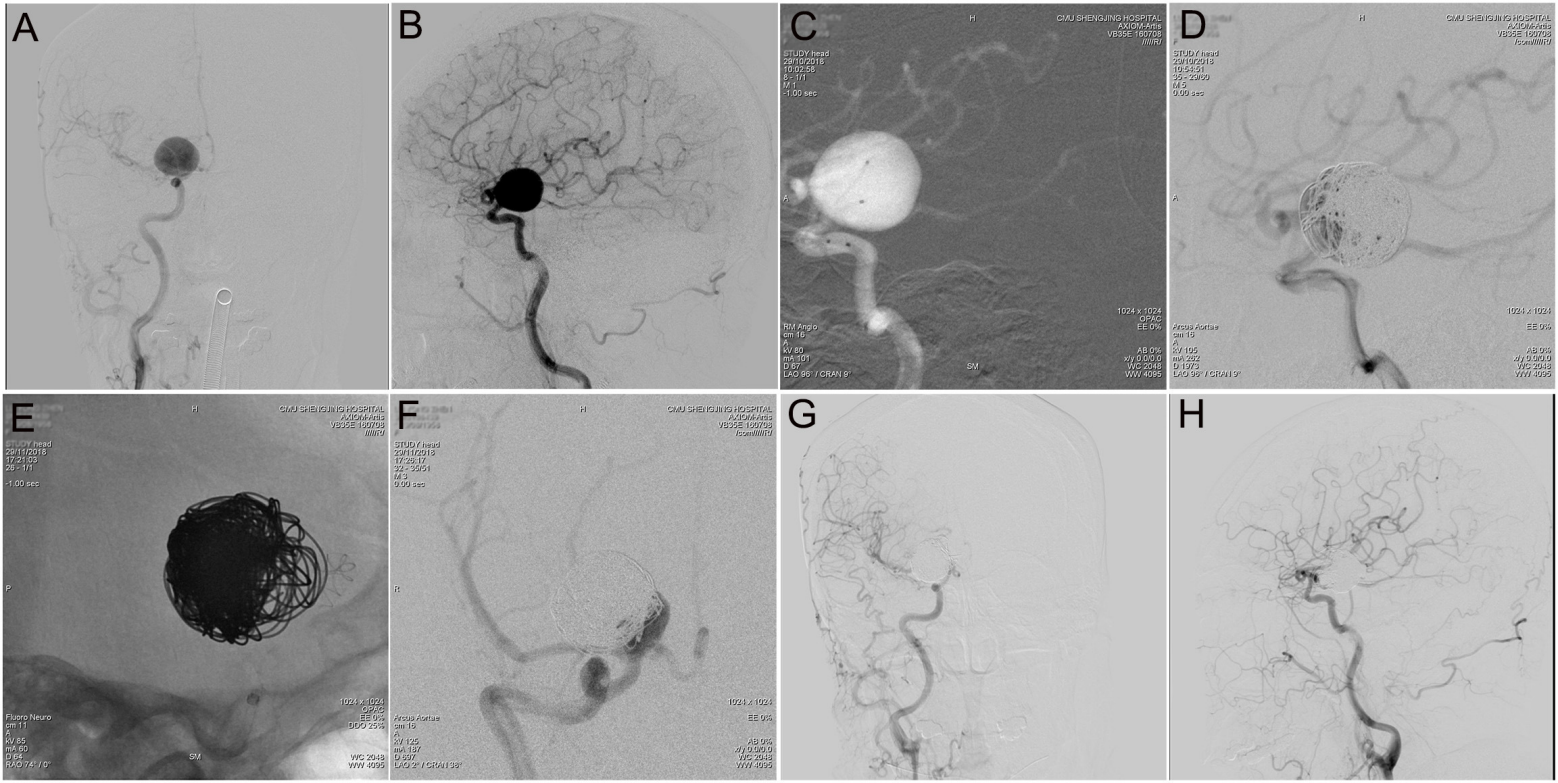
Tubridge™ treatment of a giant wide-necked aneurysm in the clinoid segment of the internal carotid artery. **(A)** DSA results shows a giant wide-necked aneurysm in the clinoid segment of the right internal carotid artery; **(B)** working angle measurement indicates an aneurysm size of approximately 21.4 mm × 20.5 mm and an aneurysm neck of 13 mm; **(C)** application of the double-microcatheter technique with spring ring embolization of the aneurysm to support the second-stage Tubridge™ release; **(D)** immediate postoperative imaging to complete most of the aneurysm embolization; **(E)** after 1 month, the second stage of Tubridge™ release is 3.0 × 25, and the stent is massaged by the microcatheter to make the stent fit the wall completely; **(F)** immediate DSA imaging shows a small amount of residual aneurysm neck; and **(G,H)** 2 years after surgery, DSA re-examination shows complete healing of the aneurysm.

Of the 92 patients, 39 were treated with the PED; a total of 42 stents were implanted, with 36 patients receiving one stent and three receiving two stents each (Figure 1). All patients in the PED group were treated with the FD combined with coils, and all PEDs were successfully implanted with a technical success rate of 100%. Five cases were not treated with guidewire massage, and 34 were treated with microwire massage to promote stent apposition, of which five were remedied with proximal balloon dilation of the stent. Fifty-three patients were treated with the TB, and a total of 58 stents were implanted; five patients had two stents implanted (Figure 2). Seven cases were not treated with guidewire massage, and 46 were treated with microwire massage to promote FD apposition, of which six were remedied with proximal balloon dilation of the FD. All TBs were successfully implanted with a technical success rate of 100%. In the TB group, five cases were treated with the FD alone and 48 were treated with the FD combined with coils. Imaging examination immediately after FD placement indicated a significant reduction in contrast filling into the aneurysm sac, and none of the cases in the PED group showed intraoperative displacement. Two patients in the TB group showed stent shortening, one showed satisfactory coverage of the aneurysm neck without additional treatment, and one received a replacement TB stent of the same diameter. Two patients in the TB group had intraoperative thrombosis, and they completely recovered after intra-arterial infusion of tirofiban and balloon angioplasty. Postoperative angiography indicated no stenosis in the artery with the aneurysm, and no occlusion of branching or penetrating arteries was observed.

### Perioperative complications

One case in the PED group showed subarachnoid hemorrhage at 36 h after operation. The patient was unconscious, voluntarily discharged from the hospital, and died 5 days later. Four patients experienced a worsening headache that improved after symptomatic and hormonal treatment, and two reported decreased muscle strength after operation; no cases of infarction were observed on CT re-examination. The patients recovered within 48 h after volume expansion and rehydration therapy, continued oral dual antiplatelet therapy, intensive lipid-lowering therapy, and anti-vasospasm therapy. No bleeding complications were observed in the TB group. In the perioperative period, three patients showed worsening headache that which improved after treatment, three showed transient loss of muscle strength, and two showed speech disorders manifesting as dysarthria. Three cases resolved completely with medication. Only one case showed mild residual hemiparesis, and MRI showed brain stem infarction in this patient. Another case showed acute hemiparesis occurring 3 d after discharge; emergency cerebral angiography re-examination indicated unimpeded flow in the artery with the aneurysm, and the patient was treated with medication. Mild residual hemiparesis was observed, and infarction of the posterior limb of the internal capsule was found on MRI.

### Postoperative follow-up

Outpatient or telephone follow-up was conducted for all patients (Table 2). During the follow-up period, two patients died, one from colon cancer and one from a car accident. In the PED group, one patient showed acute ipsilateral internal carotid artery occlusion 6 months after surgery; the patient underwent stent thrombectomy and had mild residual functional impairment. In the TB group, two patients experienced transient ischemic attacks, and the symptoms resolved after intensive lipid-lowering therapy. In the PED group, 31 patients underwent angiography re-examination; the follow-up period was 9.7 ± 3.3 months. Of these, 24 patients (77.42%) exhibited complete aneurysm occlusion, and the mRS score at the follow-up endpoint was 0.44 ± 0.31. One case showed stenosis of the parent artery. In the TB group, 42 patients underwent angiography re-examination, the follow-up period was 9.1 ± 4.4 months, with 36 patients (85.71%) exhibiting complete aneurysm occlusion, and the mRS score at the follow-up endpoint was 0.52 ± 0.28. Three cases showed stenosis, and one showed occlusion of parent artery, although the patient did not exhibit any neurological deficits.

**Table 2:** Outcome of the patients with intracranial wide-necked aneurysms who were treated with FDs.

	**Pipeline**	**Tubridge**	***P***
Follow-up (months) (median ± SD)	9.7 ± 3.3	9.1 ± 4.4	0.4759
**Treatment strategy**			
FD alone	0	5	
FD concomitant coiling	39	48	
**Number of FD were implanted**			
1	36	48	
≧2	3	5	
**Remediation strategy**			
① Microwire massage	34	46	
② Proximal balloon dilation	5	6	
**Safety**			
Technical complications	0	0	
Clinical complications			0.9037
① SAH	1	0	
② symptoms of cerebral ischemia	0	2	
**Follow-up**			
Follow-up angiography	31	42	
Aneurysm occlusion			0.3683
① Complete occlusion	24	36	
② Occlusion but residual neck	6	4	
③ Residual aneurysm	1	2	
Parent artery			0.3909
① Stenosis	0	1	
② Occlusion	1	3	
Functional at the endpoint of follow-up			
mRS score at the endpoint of follow-up	0.44 ± 0.31	0.52 ± 0.28	0.199
Mortality at the endpoint of follow up	1	1	

## Discussion

Treatment of intracranial aneurysms using FDs is a landmark advancement that has made some previously difficult and risky cases easier and safer to treat and has effectively improved long-term patient outcomes and significantly reduced the recurrence rate of aneurysms in comparison with traditional coil or stent-assisted embolization (13). The mechanism of FD treatment of intracranial aneurysms involves changing the direction of blood flow using densely distributed mesh filaments, which can decrease blood flow and induce thrombus formation in the aneurysm by significantly slowing or stagnating the blood flow in the lumen of the aneurysm. In addition, an FD provides a scaffold for endothelial growth, promoting endothelialization of the aneurysm neck and restoring arterial wall integrity (14, 15). Currently, most of the clinical evidence evaluating FDs for intracranial aneurysms has been obtained from single-arm retrospective studies or randomized trials comparing FDs with conventional therapies (4, 8, 16, 17). Comparative studies evaluating different FDs, which are important considering the increasing variety of commercially available FDs, are lacking. The PED was the first FD to be used in clinical practice and is currently the most commonly used FD. The TB, which was approved for the market in 2018, is the first Chinese-made FD. Zhou et al. (18) conducted a single-center cohort study of 28 patients with intracranial aneurysm treated with TB. Over a mean follow-up period of 9.9 months, the rate of complete aneurysm occlusion was 72%, and there were no cases of death or disability. The recent PARAT prospective multicenter trial, also conducted in China, evaluated the TB and stent-assisted coils for large/massive aneurysms with a follow-up period of 6 months and found a complete aneurysm occlusion rate of 75%, which was much higher than that in the stent-assisted coil embolization group (25%) (11).

In this study, we compared and analyzed cases of intracranial wide-necked aneurysms treated with the PED and TB in four medical centers. The rate of complete aneurysm occlusion at the end of the follow-up period was 77.42% (24/31) in the PED group and 85.71% (36/42) in the TB group; the difference was not significant (*p* > 0.05). The difference in the results of the analysis of clinical outcomes was also not significant (*p* > 0.05). These results indicate that the efficacy of the two types of FDs in the treatment of intracranial wide-necked aneurysms did not differ significantly.

FD performance was evaluated in terms of porosity, metal coverage, and mesh density. The PED's lumen is uniform in size, with a mesh size of 0.02–0.05 mm^2^ and 30–35% metal coverage within the aneurysm artery per specifications (19). The TB has a self-expanding mesh duct with an abducted end. TB's metal coverage is 35%, and the mesh size is 0.04 mm^2^ (18). When the distal and proximal diameters of the aneurysm-carrying artery are different, a larger-diameter TB can be used to ensure metal coverage and mesh density. If the PED diameter is smaller than that of the aneurysmal artery, it may be possible to increase the mesh size and to decrease the metal coverage due to the increased size (20). The transition-zone effects due to diameter differences between the aneurysmal artery and FD can be addressed by using multiple PEDs of different sizes (21).

In this study, we reviewed cases from four centers. During the perioperative period, one case of subarachnoid hemorrhage occurred in the PED group (2.56%, 1/39), and it was possibly associated with a previous history of hypertension. In contrast, two patients in the TB group developed acute ischemic stroke complications during the perioperative period (3.77%, 2/53), and MRI re-examination indicated acute brainstem infarction and internal capsule infarction. A retrospective analysis suggested that this may be related to antiplatelet drug resistance in both patients. Overall, there was no significant difference in the incidence of perioperative complications between the two groups (*p* > 0.05), indicating no difference in the safety of the two FDs in the treatment of intracranial wide-necked aneurysms.

### Radial support

Another key index of an FD is the radial support force. In comparison with conventional intracranial stents, an FD has a lower radial support force, resulting in a softer FD with better throughput, but possibly at the cost of incomplete apposition, which is detrimental to endothelialization formation, and thromboembolic complications may also occur due to insufficient radial support in the use of an FD (15, 22). In this study, the PED was found to have weak radial support and was slightly difficult to open, especially in the curved part of the vessel, necessitating repeated pushing, pulling, and jiggling to ensure complete opening; although the TB showed slightly higher relative radial support and was easier to release, no statistically significant difference was found between these two different FD radial forces in terms of their effect on passage and apposition performance. In the early cases in this group, immediate imaging results were satisfactory with pushing, pulling, and jiggling alone, although acute thrombosis within the FD or stenosis of the aneurysmal artery were observed a few min later, and the combination of microguide-forming collaterals with a microcatheter push massage and balloon dilation to the proximal end of the FD was required to reduce thromboembolic complications in some cases. Smaller changes in canal diameter after PED release along with a lower shortening rate were found in our cases; TB use also resulted in a significantly higher shortening rate due to slightly higher vascular adaptation. Therefore, with regard to device selection, both the PED and TB may be more suitable for cases with more uniform vessel diameters, while the TB with a slightly larger diameter may be more advantageous for aneurysmal arteries with large distal and proximal lumen disparities. During treatment, it is advisable to choose a slightly longer length for the TB than for the PED for the same cases.

### Use of the coil

In addition to reducing blood flow into the aneurysm leading to its thrombosis, an FD provides a framework for de novo endothelial coverage of the aneurysm neck, which completely excludes the aneurysm from circulation. Indeed, complete occlusion of the aneurysm may be largely dependent on adequate endothelialization of the FD. Using a rabbit saccular aneurysm model, Kadirvel et al. (23) demonstrated the mechanism of *de novo* endothelial coverage in the PED, with extensive endothelialization of the FD by day 7. In completely occluded aneurysms, endothelialization manifested as a thin translucent layer at 4 weeks that became thicker; by 8 weeks, 66.5 and 70% of the samples had shown complete aneurysm occlusion at 30 and 60 d, respectively. Importantly, if endothelialization did not occur, there was no aneurysmal thrombosis in any case, and when the mesh was not occluded by the overlying tissue, it was always not occluded. In this study, 94.57% (87/92) of the cases were treated with a combination of coils and an FD in the hope that the coils would slow blood flow, promote thrombosis, and facilitate aneurysm healing. Interestingly, an 80% occlusion rate was also obtained (4/5) in the limited number of cases treated with an FD alone. Therefore, for FD treatment of large intracranial wide-necked aneurysms, the selection of an appropriate stent and appropriate release technique to optimize metal coverage and mesh density may be more important.

### Limitations of the study

The main limitation of this study was the selection bias associated with different centers and physicians. The study duration was long, and the study was conducted at four centers; thus, the clinicians lacked sufficient clinical experience in most of the early cases; the strategies and techniques used in the procedures changed over time; and some of the complications may have been due to physician inexperience rather the instrumentation. Moreover, the imaging follow-up modality and time points were selected at the discretion of the patient and the assessment of outcomes was not blinded, which may have resulted in partial investigator bias. In addition, data collection and analysis were performed retrospectively, because of which the possibility of incomplete data cannot be ruled out.

## Conclusion

Both the PED and TB are safe and effective in the treatment of large intracranial wide-carotid aneurysms, with no significant difference in the rate of complete aneurysm occlusion on angiography (77.42 vs. 85.71%, *p* > 0.05) or rate of perioperative complications (2.56 vs. 3.77%, *p* > 0.05) between the two. The TB shows advantages related to vascular lumen adaptability and release but is associated with has a higher incidence of shortening, while the PED shows the most reliable evidence at present and is still being upgraded. The technical characteristics of the two FDs may confer specific advantages in various indications, although large, prospective, controlled trials are needed to compare them and to determine the best indications for both.

## Data availability statement

The original contributions presented in the study are included in the article/supplementary material, further inquiries can be directed to the corresponding authors.

## Ethics statement

The studies involving human participants were reviewed and approved by the Institutional Review Board of Shengjing Hospital of China Medical University. The patients/participants provided their written informed consent to participate in this study.

## Author contributions

ZL, FY, YG, and YX contributed to the study design. HC, FY, YG, YX, MW, and ZL performed the clinical follow-up. HC, JL, YL, KF, CL, and ZL performed the literature search, data collection, and drafted the manuscript. ZL, FY, YG, and YX contributed to data analysis and interpretation. HC and ZL contributed to editing and revision of the manuscript. All authors contributed to the article and approved the submitted version.

## Funding

This work is supported by grants from the National Natural Science Foundation Project of Liaoning Province (2019-ZD-0757), Joint fund of Science and Technology Department of Liaoning Province and State Key Laboratory of Robotics of China (2020-KF-12-06), Shenyang Science and Technology Bureau (No. 19-112-4-044, 21-172-9-17), and 345 Talent Project fund of Shengjing Hospital and Young Talents Program of China Medical University (M270).

## Conflict of interest

The authors declare that the research was conducted in the absence of any commercial or financial relationships that could be construed as a potential conflict of interest.

## Publisher's note

All claims expressed in this article are solely those of the authors and do not necessarily represent those of their affiliated organizations, or those of the publisher, the editors and the reviewers. Any product that may be evaluated in this article, or claim that may be made by its manufacturer, is not guaranteed or endorsed by the publisher.
